# Mathematical Modeling of p53 Pathways

**DOI:** 10.3390/ijms20205179

**Published:** 2019-10-18

**Authors:** Eunjung Kim, Jae-Young Kim, Joo-Yong Lee

**Affiliations:** 1 Graduate School of Analytical Science and Technology (GRAST), Chungnam National University, Daejeon 34134, Korea; eunjung.kim@cnu.ac.kr (E.K.); jaeyoung@cnu.ac.kr (J.-Y.K.); 2 Korea Basic Science Institute, Daejeon 34133, Korea

**Keywords:** p53 dynamics, mathematical modeling, DNA damage response, metabolism

## Abstract

Cells have evolved balanced systems that ensure an appropriate response to stress. The systems elicit repair responses in temporary or moderate stress but eliminate irreparable cells via apoptosis in detrimental conditions of prolonged or severe stress. The tumor suppressor p53 is a central player in these stress response systems. When activated under DNA damage stress, p53 regulates hundreds of genes that are involved in DNA repair, cell cycle, and apoptosis. Recently, increasing studies have demonstrated additional regulatory roles of p53 in metabolism and mitochondrial physiology. Due to the inherent complexity of feedback loops between p53 and its target genes, the application of mathematical modeling has emerged as a novel approach to better understand the multifaceted functions and dynamics of p53. In this review, we discuss several mathematical modeling approaches in exploring the p53 pathways.

## 1. Introduction

Mutations in the tumor suppressor p53 gene have been frequently linked to human cancers [1]. p53 induces the transcription of genes involved in cell cycle control and apoptosis in response to cellular stresses, such as DNA damage [2]. p53 also regulates additional biological processes including autophagy, metabolism, and mitochondrial physiology [3,4,5,6,7,8]. These multifaceted functions of p53 are tuned by its simultaneous interaction with various target genes. An excellent recent review provided a comprehensive list of hundreds of p53 target genes from not only individual gene studies but also high throughput analyses [9]. Here, we highlight a few p53 target genes involved in different biological processes (Table 1), which were identified as high-confidence p53 genes by gene ontology enrichment analysis in [9]. For example, in the cell cycle arrest process, p53 activates cyclin dependent kinase inhibitor 1A (*CDKN1A/p21*) that inhibits cyclin dependent kinases and proliferating cell nuclear antigen (*PCNA*) [10]. The p53 target genes involving cell apoptosis include B-cell lymphoma 2 (BCL-2) family members such as Bcl2-associated X protein (BAX) [11] and p53 upregulated modulator of apoptosis [12], which control cytochrome c release from the mitochondria. p53 also regulates cellular metabolism. It inhibits glycolysis via inducing TP53 inducible glycolysis and apoptosis regulator (TIGAR) that reduces fructose-2-6-bisphosphate [13]. The complexity of the p53 network has made it challenging to gain comprehensive insights into it, motivating the development and application of various quantitative approaches for exploring this network.

Mathematical modeling is a valuable tool in effort to understand complex biological phenomena, especially cancer [14] and cell signaling networks [15,16]. Modeling is an abstract and logical representation of biological processes, such as the interaction of many molecules in a signaling network. It is capable of elucidating critical components of the systems under observation and quantifying their behavior under given conditions. Furthermore, mathematical modeling allows us to test many hypothetical scenarios that are impossible to recreate in experiments and generates testable predictions. Its integration with a detailed understanding of biology can provide new insights into the mechanisms of biological processes [17]. Mathematical approaches can be applied to understanding p53 functions as well as dynamical interactions between p53 and other genes in response to various stresses, such as DNA damage and metabolic stress. To explain time-dependent kinetics of the p53 signaling network, the ordinary differential equation (ODE) modeling approach was utilized. The ODE describes the rate of change of variables (e.g., total p53 protein level) with respect to time as a function of other variables (e.g., the concentration of other proteins). Given model parameter sets, such as production rate, degradation rate, and binding or dissociation rate, ODE models can accurately predict temporal profiles of the p53 and explain how p53 dynamics influence cell fate decisions, such as survival and death. To understand spatial patterns of p53, partial differential equation (PDE) modeling can be utilized. The PDE method explains the evolution of variables in space and time. All the modeling approaches discussed so far are deterministic and assume a continuous time scale. Cellular automata (CA) describes discrete states (e.g., on or off) of an individual agent in a grid point in a discrete time scale. The modeling approach can explain the spatial dependence of one variable on its neighbors, since this approach describes the change of a variable as a function of the states of its neighbors at discrete time points. Other modeling approaches such as Markov process or stochastic random walk simulation were used to account for stochastic fluctuations of p53. 

The goal of this review is to offer a comprehensive view of various mathematical modeling approaches that have been employed to explore the tumor suppressive roles of p53. We summarize mathematical modeling approaches focusing on three key biological contexts regulated by p53—DNA damage response, cellular metabolism and mitochondrial physiology. 

## 2. p53 Dynamics in DNA Damage Response

### 2.1. Biological Background for Mathematical Model Development

Cells are continuously exposed to intrinsic (e.g., DNA replication stress) and extrinsic DNA damaging stressors (e.g., UV, radiation, chemical carcinogens), which cause abnormal chemical modifications in the DNA structure, leading to genomic abnormality if not correctly repaired [18]. Failure in maintaining genome integrity is closely linked to emergence of hallmarks of cancer. p53 has been described as “the guardian of the genome” due to its critical roles in DNA damage response. 

In normal and unstressed conditions, low physiological levels of p53 are maintained by a p53 negative regulator, E3 ubiquitin-protein ligase Mouse double minute 2 homolog (Mdm2), that promotes rapid p53 degradation [19,20]. Mdm2 production is also controlled by p53 [21]. DNA damage, however, disturbs this balance between p53 and Mdm2, leading to elevation of p53 and upregulation of p53 target genes involved in the cell cycle, DNA repair, or apoptosis [9,22]. Interestingly, various temporal profiles of p53 have been observed. For example, oscillatory p53 protein level change has been observed in both in vitro [23,24] and in vivo [25]. DNA breaks caused by ionizing radiation generates a series of pulse responses of p53 [26] while ultraviolet (UV) radiation induces a single p53 pulse [27]. Sustained p53 levels at a peak level was generated after addition of small molecule Mdm2 inhibitor under DNA damage condition [26]. 

### 2.2. Mathematical Models of p53 in the DNA Damage Response

**Figure 1:**
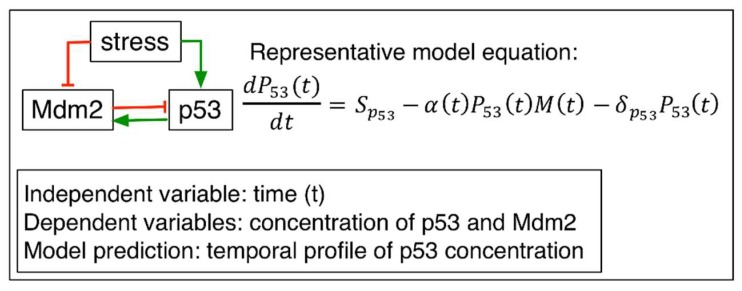
An ODE model developed by Lev Bar-Or et al [24]. Figure and equation adapted with permission from [24]. Copyright 2000 National Academy of Sciences of the United States of America. Left: model diagram, right: a representative model equation that describes p53 protein changes with respect to time as a function of source, degradation by Mdm2 (M(t)), and natural decay. Green line: activation or production, red line: inhibition or degradation.

A number of ODE models have been used to describe the complex feedback among the large number of molecules involved in the p53 network [28,29]. Some mathematical modeling efforts have predicted distinct temporal behaviors of p53 as damped and undamped regular response. Initial models focused on the core negative feedbacks between p53 and Mdm2. The cellular concentration of p53 and Mdm2 was modeled using kinetic equations. For example, Lev Bar-Or and colleagues developed the following system of ODEs (Figure 1) [24]. In the model, p53 protein level was governed by an ODE $\frac{dP_{53}\left(t\right)}{dt}=S_{p_{53}}-\alpha \left(t\right)P_{53}\left(t\right)M\left(t\right)-\delta_{p_{53}}P_{53}\left(t\right)$, which described the change of the concentration of p53 as a function of p53 production (source: $S_{p53})$, degradation by Mdm2 (M(t)) and its natural decay (rate: $\delta_{p53})$. The degradation of p53 by Mdm2 was explained by another time-dependent function α(t) ($\alpha \left(t\right)=\alpha_{basal}-\left[\kappa \times E\left(t\right)-\kappa_{0}\left(t\right)\right],\kappa_{0}\left(t\right)=threshold$), where $\alpha_{basal}$ represented a degradation rate controlling the basal level of p53, E(t) indicated stress signal, and κ indicated inhibition of degradation by stress signal. The threshold ($\kappa_{0}\left(t\right))$ was introduced to explain a damping effect on this inhibition ($\frac{d\kappa_{0}\left(t\right)}{dt}=-\beta \kappa_{0}\left(t\right)E\left(0\right),\;\kappa_{0}\left(0\right)=\beta E\left(0\right)).\;$ The Mdm2 protein dynamics was modeled by another ODE equation $\frac{dM\left(t\right)}{dt}=S_{M}+p_{max}I{\left(t\right)}^{n}/\left(K+I{\left(t\right)}^{n}\right)-\delta_{M}M\left(t\right)$, where $S_{M}$ represented p53-independent translation and transcription and $\delta_{M}$ is a natural decay rate of Mdm2. The second term in this equation described p53-dependent Mdm2 translation and transcription. This ODE model predicted a series of damped oscillatory dynamics of p53 in response to γ irradiation, which was confirmed by subsequent experiments [24]. In addition to the p53-Mdm2 feedback loop, several other proteins were included in other ODE models. Sun et al. included ataxia telangiectasia mutated (ATM), wild-type p53-induced phosphatase 1 (Wip1), and p21 in addition to the p53-Mdm2 core feedback loop [30]. Specifically, they included ATM activation of p53 and Mdm2. p53 can activate p21 and Wip1, a negative regulator of both ATM and p53. A system of ODE equations was developed to model these interactions. The model predicted that basal DNA double strand breeaks during normal cell cycle progression triggers spontaneous pulses of p53 not sufficient for p21 induction. Only extensive DNA damage generage synchornous p53 pulses that induces high level of p21.

More efforts were made to describe p53 dynamics during DNA damage response [26,31,32,33,34]. In particular, Purvis *et al*. utilized an integrated approach of experiments and mathematical modeling to develop a method that can switch pulsed p53 dynamics to a sustained p53 at a peak level in DNA damage response [26]. The mathematical model explained the feedback loop of stress signal involving inactive p53, active p53, Mdm2, and Wip1 by delayed differential equations incorporating time delayed induction of Mdm2 and Wip1 by p53. The model assumed that inactive p53 was produced by a constant source in a cell, degraded by Mdm2 following a Hill-type relation, decayed at some natural decay rate. DNA damage signal converted inactive p53 into active p53 at some rate, dependent on the magnitude of the damage signal. The active p53 was degraded by Mdm2. In the model, Mdm2 was produced by active p53, reduced by DNA stress signal, and decayed at some natural decay rate. The dynamics of Wip1 was modeled as a function of active p53 induction and natural decay. Finally, DNA stress signal was modeled using a time-dependent DNA damage source, natural decay, and threshold inhibition. Using this model, they identified a treatment sequence of a small molecule Mdm2 inhibitor, which could change p53 dynamics from pulsed to a sustained level. The study demonstrated that the change of p53 dynamics led to different sets of target gene activation as well as distinct cell fates, such as DNA repair or senescence [26]. p53 targets associated with cell cycle control and DNA repair such as CDKN1A, growth arrest and DNA-damage inducible protein (GADD45A), Mdm2 and protein phosphatase 1D (PPM1D) displayed an oscillatory response like p53 protein. When p53 dynamics was switched to a sustained level, some targets such as Mdm2 and CDKN1A increased to a sustained level. Interestingly, p53-targets associated with apoptosis and senescence such as apoptotic protease activating factor 1 (APAF1), BAX, promyelocytic leukemia (PML) and yippee-like 3 (YPEL3) were not activated by p53 pulse. The genes were only induced by a sustained p53 level. The study also investigated the impact of p53 dynamics on cell fate decisions and demonstrated that pulsing p53 dynamics led to recovery from DNA damage, whereas cells with sustained p53 levels frequently underwent senescence [26].

p53-dependent cell fate decision was further investigated by different groups. By incorporating apoptosis-related genes into the core feedback loops in p53 network, some models predicted p53 as a driver of cell fate transition from cell cycle arrest to apoptosis [35,36,37]. Mathematical models that integrated ODE models for cell cycle and DNA damage network predicted the role of p53 in determining immediate or sustained cell cycle types [38,39]. In particular, the Tyson group developed a comprehensive mathematical framework describing principles of kinetics in cell apoptosis that captures the following three key dynamical features: signal threshold for cell death, a time delay between the signal and response, and irreversible commitment to cellular breakdown for apoptosis [35]. Using this framework, they proposed a process of p53 responses to DNA damage by first eliciting cell cycle arrest, followed by damage repair and cell death. The model predicted the order of protein expressions and posttranslational modifications during the cell cycle process, which were in good agreement with previously reported experimental observations. 

Lahav and collogues reported heterogeneous p53 dynamics at a single cell level [23]. Geva-Zatorsky et al. also observed variability of p53 dynamics in isogenic cells under similar conditions following DNA damaging γ radiation [40]. They found differential amplitude and period of the p53 pulses at a single cell level. Further, they developed several models of p53-Mdm2 feedback loops and identified the source of this single cell variability in the oscillations, with the noise in protein production rates contributing the most. On the contrary, Batchelor and colleagues demonstrated that different stressors induced different p53 feedback loops, thereby triggering different temporal profiles of p53 in single cells [27]. They observed that UV irradiation induced a single graded pulse of p53 while γ irradiation caused excitable p53 dynamics. Using a mathematical model integrated with single cell experimental data, they identified mechanisms for the difference. p53-Mdm2 feedback loop by ATR was responsible for the graded profile, while feedback from Wip1 to ATM induced excitable behavior. In addition, other mathematical models proposed to understand the heterogeneity in this network. Moore and colleagues integrated p53-Mdm2 core feedback loops with miRNA-mediated positive feedback loops, such as those involving miR-192, miR-34a, and miR-29a [41]. They reproduced single cell level variability of p53 dynamics and predicted that miRNA repression changes p53 dynamics significantly. 

**Figure 2:**
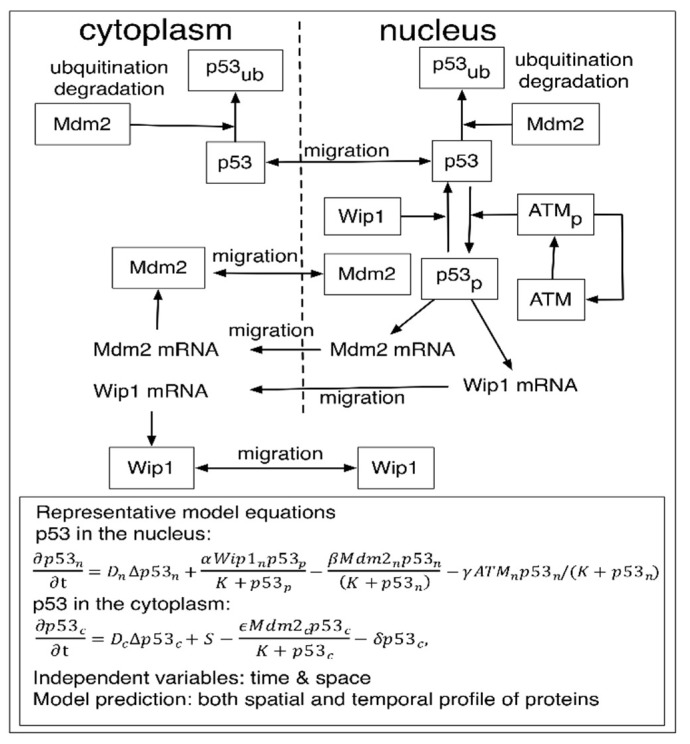
A PDE model developed by Elias et al. [42] Figure and equations adapted with permission from [42] Copyright 2014 Physical Biology. The model diagram explains p53, Mdm2, Wip1 dynamics both in the nucleus and the cytoplasm. Representative partial differential equations that described change of p53 with respect to time in the nucleus as well as in the cytoplasm.

The spatial or structural variation between individual cells was investigated by PDE models [42]. Elias and colleagues developed a PDE model describing p53 network in the nucleus and the cytoplasm. Specifically, the feedback loops between p53, Mdm2, ATM, and Wip1 in the nucleus and the cytoplasm were described by a system of partial differential equations (Figure 2) [42,43]. For example, the dynamics of p53 in the nucleus ($p53_{n})$ was explained by $\frac{\partial p53_{n}}{\partial t}=D_{n}\Delta p53_{n}+\frac{\alpha Wip1_{n}p53_{p}}{K+p53_{p}}-\frac{\beta Mdm2_{n}p53_{n}}{\left(K+p53_{n}\right)}-\gamma ATM_{n}p53_{n}/\left(K+p53_{n}\right)$, where the subscript n indicates the nucleus, $D_{n}$ indicates a diffusion rate of $p53_{n}\;$ and $p53_{p}$ represents phosphorylated p53. In the above equation, the second term represents an increase of p53 level in the nucleus by phosphorylated p53 and Wip1, and the third term represents decay of $p53_{n}\;$by Mdm2 located in the nucleus. The last term explains an increase of p53 in the nucleus by ATM. Similarly, the dynamics of p53 in the cytoplasm ($p53_{c})$ was described by $\frac{\partial p53_{c}}{\partial t}=D_{c}\Delta p53_{c}+S-\frac{\epsilon Mdm2_{c}p53_{c}}{K+p53_{c}}-\delta p53_{c},\;$ where $D_{c}$ indicates a diffusion rate of $p53_{c}$, S represents a source term, and δ represents a natural decay rate of $p53_{c}$. The second term explains the degradation of $p53_{c}$ by Mdm2 in the cytoplasm. They explained the dynamics of Mdm2, ATM, Wip1 by applying a similar modeling approach. The PDE model successfully simulated the observed p53 oscillation due to various stresses and concluded the structural variation between individual cells could be another potential source for the variability of p53 in single cells.

### 2.3. Mathematical Modeling of p53 Interaction with Other Tumor Suppressors 

In addition to undamped regular oscillations of p53 in the context of DNA damage response, mathematical models describing interactions between the core p53 feedback model and other tumor suppressor proteins were also developed. ODE models involving ATM-Mdm2-p53-PTEN-AKT feedback was developed to describe the two-phase dynamics of p53 [31,44]. In addition to the core ATM-Mdm2-p53 feedback loop, these models assumed that p53 could activate phosphatase and tensin homolog (PTEN), which inhibits protein kinase B (AKT), as an Mdm2 activator. These models demonstrated that the concentration of PTEN would be critical for transitioning to the second phase activation of p53, which is sufficient for inducing cell death [31,44]. 

A tumor-suppressive network model for mitogenic and oncogenic signals composed of three different modules, such as p53 induction, proliferation, and apoptosis, explained how cells make cell fate decisions, such as those for growth, death, and cell cycle arrest [45]. The study by Hat et al. simulated a stochastic process (Markov process) as well as deterministic ODEs composed of three different modules including the core p53 feedback, cell cycle control, and apoptosis modules [46]. The core module included interactions between ATM, Mdm2, p53, Wip1, AKT, and PTEN. The cell cycle module contains interaction between p53, p21, Cyclin E, retinoblastoma protein (Rb1), and transcription factor (E2F1), while apoptosis module was controlled by feedback between p53, Bax, Bcl-2, AKT, and Caspase. It is worth to note that this study described a detailed description of p53 network by including site-specific phosphorylation of each molecule. For example, they described the phosphorylation of p53 by ATM at Ser15 for cell cycle arrest in response to DNA damage (p53_a_) and the phosphorylation of p53 at Ser46 for apoptosis (p53_b_). This study demonstrated the ratio of Wip1 to PTEN being responsible for diverse p53 dynamics, an oscillation of p53_a_, and a fast transition to a peak level of p53_b_ in cancer cells.

## 3. p53 and Metabolism 

### 3.1. Experimental Findings Regarding the Roles of p53 in Metabolism

In addition to its role in the DNA damage response, p53 is involved in cellular metabolism [4,6,7,8]. Metabolism is the process through which the nutrients taken up by cells are metabolized to produce building blocks for biosynthesis and generate energy. Changes in metabolic activity are typically observed in the tumor initiation and progression stages, which lead to the rapid expansion and survival of cancer cells under abnormal tumor microenvironmental conditions. Many experimental studies demonstrated that p53 mediates the regulation of metabolic activity through multiple mechanisms (molecular mechanisms reviewed in [5,6]). A well-known role of p53 includes regulation of autophagy-related genes such as 5' AMP-activated protein kinase (*AMPK*) and tuberous sclerosis complex 2 (*TSC2*) under nutrient deprived conditions, as well as modulation of the expression of several glycolysis related genes such as Hexokinase 2 (*HK2*) [47] and TP53 inducible glycolysis and apoptosis regulator (*TIGAR*) [13]. More recent findings demonstrate the role of p53 in modulating glucose uptake by balancing intracellular glucose level. A study utilizing multiple cell lines showed that p53 might reduce expression of glucose transporters (e.g., GLUT-1 and GLUT-4) to prevent further glucose uptake in case of high intracellular glucose levels [48]. The p53 family member, TAp63, appeared to promote glucose uptake when the cellular glucose level is low [49], although intermediate molecular mechanisms are yet to be identified. In addition, the Flores group showed that TAp63 played important roles in regulating energy metabolism. They observed that TAp63 -/- mice develop obesity, glucose intolerance, and insulin resistance. Mitochondrial dysfunction and defects in fatty acid oxidation were also observed in this mouse model [49]. 

### 3.2. Mathematical Models of Cancer Metabolism 

Despite ample evidence suggesting the interaction of p53 with key molecules involved in metabolism, not many mathematical models have directly addressed its role, possibly due to the lack of temporal dynamics data from experimental model systems. A few mathematical models were developed to describe the p53 network in metabolism, especially under nutrient-deprived conditions. The model predicted the temporal behavior of autophagy-related genes as well as their interaction with p53 [45,50,51]. In particular, a comprehensive model developed by Liu et al. included a detailed interaction network model of autophagy and predicted that p53 might regulate cell fate transition from autophagy to apoptosis [45,50,51]. Yu et al. developed a systems biology framework explaining the regulatory principles of glycolysis and oxidative phosphorylation [51]. Their regulatory network model showed that cancer cells have a hybrid state including both oxidative and glycolytic states, due to high reactive oxygen species production and oncogene activation. These hybrid phenotypes of cancer cells appear to promote metabolic plasticity to confer cancer cells adaptability to various microenvironments.

Various mathematical models were developed to describe other aspects of cancer metabolism. These models explained not only intracellular metabolic changes in the tumor, but also their effect on tumor invasion and drug resistance. Extensive efforts were made to establish metabolic flux balance models that describe the steady-state rate of metabolic biochemical reactions in cancer cells [52]. The inclusion of reaction rates and corresponding parameters provided improved predictions of glycolysis in cancer progression [53,54]. To explain spatial-temporal variations of the metabolites, a reaction-diffusion PDE model was also developed [55]. Other PDE models were applied to understanding the effect of the tumor microenvironment on tumor metabolic changes [56,57]. A different modeling approach, hybrid cellular automata (HCA) predicted the impact of intercellular metabolic variability on tumor progression and drug resistance [58]. HCA modeling approach combines a PDE model and a cellular automata model. A PDE model describes the diffusion of a chemical such as nutrient, oxygen and acid, while a cellular automaton model (CA) explains individual cell behavior (phenotype) on a grid point. Cell phenotypes typically include cell death, migration, and proliferation, which is determined by microenvironmental factors as well as cell intrinsic machineries such as signaling pathways or metabolic pathways. Robertson-Tessi et al. developed an HCA model of cancer cell metabolism which described a detailed description of ATP production in each cell [58], where cell fate was determined by the metabolic state of each cell. 

## 4. Mathematical Models of Mitochondrial Physiology

### 4.1. Biological Background 

Mitochondria are dynamic populations within the cell undergoing continuous merging (fusion) and division (fission) and act as the powerhouse of the cell by regulating various processes, such as oxidative phosphorylation, biogenesis, thermogenesis, production of lipids and amino acids, and induction of apoptosis. Aberrant mitochondrial function is linked to several hallmarks of cancer conferring a survival advantage [59]. For example, mitochondrial DNA mutations are often observed in cancer, leading to abnormal energy metabolism and increase in reactive oxygen species. The increase of mitochondrial DNA mutations appears to be associated with the loss of p53. Several studies have demonstrated that p53 contributes to mitochondrial genome stability [60,61] and enhances the accuracy of DNA synthesis [62,63]. The Hwang group demonstrated that p53 protein maintains the mitochondrial genome in response to both intrinsic and extrinsic factors such as reactive oxygen species. In response to stress, p53 translocates to mitochondria and physically interacts with both the DNA and mitochondria polymerase γ. Subsequent studies have reported additional roles of p53 in mitochondrial DNA regulation, leading to the emerging paradigm of p53 as a guardian of the mitochondrial genome [62,63]. p53 is also involved in mitochondrial length control and cycling of fusion and fission [64], which is essential for normal mitochondrial function and mutation-free mitochondrial DNA synthesis. In this session, we describe several mathematical modeling approaches describing mitochondrial DNA maintenance, as well as dynamical process of mitochondrial fusion and fission.

### 4.2. Mathematical Models of Mitochondrial Physiology

Several mathematical models have described mitochondrial physiology, although the multifaceted roles of p53 in regulating mitochondrial physiology have not been fully incorporated yet. A quantitative analysis of the mitochondrial network clusters within cells was developed to identify structural parameters defining the mitochondrial network [65]. In this study, Zamponi et al. analyzed mitochondrial networks using confocal microscopy images, determined the underlying structural parameters based on these images, and then calculated mitochondrial cluster mass and degree (number of nearest neighbors), which were used to define the mitochondrial network configuration. ODE models described mitochondrial quality control such as mitochondrial DNA synthesis and deletion [66,67], and predicted consequences of this DNA deletion. The model showed that shorter size of DNA has a selection advantage in promoting additional deletion mutations. The biogenesis of mitochondria into a large-scale stable organization within the cell was simulated by using both a mean field ODE model and the agent-based modeling approach [68]. Stochastic simulation was applied to model the dynamic mitochondrial fusion and fission process. Patel et al. developed a stochastic spatiotemporal model of fusion-fission [64,69]. They described local interactions between neighboring mitochondria and their reorganization. To define mitochondrial population health, they utilized the asymmetry of electrochemical potential across the inner membrane. Using the model, they showed that mitochondrial density did not affect mitochondrial population health, as long as a minimum basal rate of fusion was maintained. The fusion rate was predicted to be enhanced by actively regulating mitochondrial motility. Another stochastic simulation was applied to explain other roles of fusion-fission cycles in mitochondrial DNA maintenance [70]. Here, Tam et al. developed a stochastic model (random walk model) to simulate the distribution of both wild type and mutant DNA sequences in mitochondria. The DNA turnover such as replication, degradation, and DNA mixing through mitochondrial fusion and fission was modeled as a random event with a probability proportional to a pre-defined time step size as well as a propensity function. To describe mitochondria spatial distribution in a cell, a cell in the model was compartmentalized into several regions based on single mitochondrial traveling distance. The Gillespie algorithm was used to simulate the stochastic events of both mitochondrial DNA turn over and movement [71]. The model simulations with various fusion-fission rates demonstrated that a slower fusion-fission rate leads to increased stochasticity in the mitochondrial DNA mutation burden in a tissue.

## 5. Future Perspectives

Several important questions remain despite remarkable progress in mathematical modeling of the p53 pathway. For example, quantitative approaches that help to explain p53 in regulating metabolism and mitochondrial physiology are still lacking. The lack of mathematical modeling in these aspects is, in part, due to a lack of detailed temporal data of p53 in these areas. Obtaining experimental data is necessary to develop mathematical models that can accurately predict p53 network dynamics since the ability of a mathematical to predict a system’s behavior depends on mathematical model parameterization. It also remains unclear how p53 dynamics is changing upon p53 mutations. It is known that newly established interactions of mutant p53 with other cellular proteins can deprive cells of tumor-suppressive response and promote cancer development [72]. The detailed p53 dynamics in these newly established networks, however, are still unknown. Mathematical models of these newly interactions have the potential to bridge gaps in our efforts to comprehensively characterize p53 dynamics in both normal and cancer development conditions. In addition, the development of open resource tools for mathematical models of p53 network like p53 biological resource (https://p53.fr for p53 biological resource) will further assist p53 modeling community.

## 6. Conclusions

The p53 pathway has captivated the attention of both experimental and mathematical modeling research communities and is one of the most well-studied pathways. In addition to its classic role as a genome guardian under genotoxic stress, increasing evidence indicates p53 is associated with other cellular processes such as regulation of metabolism and mitochondrial physiology. In this review, we discussed various mathematical modeling approaches simulating p53 networks, focusing on three areas – classical p53-medated DNA damage response, roles of p53 in cellular metabolism and mitochondrial dynamics. These mathematical models have provided useful insights to better understand the multifaceted role of p53. To develop mathematical models, biological knowledge about p53 network or new experimental measurements were first considered. These data typically served to derive mathematical model assumptions. Then, appropriate mathematical modeling approaches that can represent both observed experimental measurements and main questions were selected. Mathematical model simulations generated novel hypotheses, which were tested and confirmed in subsequent experiments. Further studies of integrating mathematical models with systematic measurements will be useful for unveiling new p53 mechanisms.

## Figures and Tables

**Table 1:** A few p53 target genes in cell cycle arrest, apoptosis, and DNA repair (a complete list can be found in [9]).

Biological Process	p53 Target Genes
Cell cycle arrest	*GADD45A, BTG2, SFN, CDKN1A*
Apoptosis	*BAX, APAF1, AEN, FAS, PERP, TRIAP1, BBC3, PMAIP1, SUSD6*
DNA repair	*XPC, PCNA, POLH, RRM2B*

Note: Key p53 target genes referred as high confidence genes in [9], where high confidence was defined as a p53-activated genes identified in at least three high throughput studies. Full gene names are listed in the Abbreviations section below.

## Abbreviations

GADD45A	Growth arrest and DNA damage inducible alpha
BTG2	B-cell translocation gene 2
SFN	Stratifin
CDKN1A	Cyclin dependent kinase inhibitor 1A
BAX	Bcl2 associated protein X
APAF1	Apoptotic protease activating factor 1
AEN	Apoptosis enhancing nuclease
FAS	Fas cell surface death receptor
PERP	p53 apoptosis effector related to PMP-22
TRIAP1	TP53 regulated inhibitor of apoptosis 1
BBC3	Bcl-2 binding component 3
PMAIP1	Phorbol 12 Myristate 13 acetate induced protein 1
SUSD6	Sushi domain containing 6
XPC	Xeroderma pigmentosum
PCNA	Proliferating cell nuclear antigen
POLH	DNA polymerase eta
RRM2B	Ribonucleotide reductase regulatory TP53 inducible subunit M2B.

## References

[1] Kandoth C., McLellan M.D., Vandin F., Ye K., Niu B., Lu C., Xie M., Zhang Q., McMichael J.F., Wyczalkowski M.A. (2013). Mutational landscape and significance across 12 major cancer types. Nature.

[2] Riley T., Sontag E., Chen P., Levine A. (2008). Transcriptional control of human p53-regulated genes. Nature Rev. Mol. Cell Biol..

[3] Berkers C.R., Maddocks O.D., Cheung E.C., Mor I., Vousden K.H. (2013). Metabolic regulation by p53 family members. Cell Metab..

[4] Bieging K.T., Mello S.S., Attardi L.D. (2014). Unravelling mechanisms of p53-mediated tumour suppression. Nat. Rev. Cancer.

[5] Li T., Kon N., Jiang L., Tan M., Ludwig T., Zhao Y., Baer R., Gu W. (2012). Tumor suppression in the absence of p53-mediated cell-cycle arrest, apoptosis, and senescence. Cell.

[6] Napoli M., Flores E.R. (2017). The p53 family orchestrates the regulation of metabolism: Physiological regulation and implications for cancer therapy. Br. J. Cancer.

[7] Vousden K.H., Ryan K.M. (2009). p53 and metabolism. Nat. Rev. Cancer.

[8] Floter J., Kaymak I., Schulze A. (2017). Regulation of Metabolic Activity by p53. Metabolites.

[9] Fischer M. (2017). Census and evaluation of p53 target genes. Oncogene.

[10] El-Deiry W.S., Tokino T., Waldman T., Oliner J.D., Velculescu V.E., Burrell M., Hill D.E., Healy E., Rees J.L., Hamilton S.R. (1995). Topological control of p21WAF1/CIP1 expression in normal and neoplastic tissues. Cancer Res..

[11] Miyashita T., Reed J.C. (1995). Tumor suppressor p53 is a direct transcriptional activator of the human bax gene. Cell.

[12] Nakano K., Vousden K.H. (2001). PUMA, a novel proapoptotic gene, is induced by p53. Mol. Cell.

[13] Bensaad K., Tsuruta A., Selak M.A., Vidal M.N., Nakano K., Bartrons R., Gottlieb E., Vousden K.H. (2006). TIGAR, a p53-inducible regulator of glycolysis and apoptosis. Cell.

[14] Altrock P.M., Liu L.L., Michor F. (2015). The mathematics of cancer: Integrating quantitative models. Nat. Rev. Cancer.

[15] De Jong H. (2002). Modeling and simulation of genetic regulatory systems: A literature review. J. Comput. Biol..

[16] Eungdamrong N.J., Iyengar R. (2004). Modeling cell signaling networks. Biol. Cell.

[17] Gatenby R. (2012). Perspective: Finding cancer’s first principles. Nature.

[18] Lindahl T., Barnes D.E. (2000). Repair of endogenous DNA damage. Cold Spring Harb. Symp. Quant. Biol..

[19] Haupt Y., Maya R., Kazaz A., Oren M. (1997). Mdm2 promotes the rapid degradation of p53. Nature.

[20] Kubbutat M.H., Jones S.N., Vousden K.H. (1997). Regulation of p53 stability by Mdm2. Nature.

[21] Barak Y., Juven T., Haffner R., Oren M. (1993). mdm2 expression is induced by wild type p53 activity. EMBO J..

[22] Verfaillie A., Svetlichnyy D., Imrichova H., Davie K., Fiers M., Kalender Atak Z., Hulselmans G., Christiaens V., Aerts S. (2016). Multiplex enhancer-reporter assays uncover unsophisticated TP53 enhancer logic. Genome Res..

[23] Lahav G., Rosenfeld N., Sigal A., Geva-Zatorsky N., Levine A.J., Elowitz M.B., Alon U. (2004). Dynamics of the p53-Mdm2 feedback loop in individual cells. Nat. Genet..

[24] Lev Bar-Or R., Maya R., Segel L.A., Alon U., Levine A.J., Oren M. (2000). Generation of oscillations by the p53-Mdm2 feedback loop: A theoretical and experimental study. Proc. Natl. Acad. Sci. USA.

[25] Hamstra D.A., Bhojani M.S., Griffin L.B., Laxman B., Ross B.D., Rehemtulla A. (2006). Real-time evaluation of p53 oscillatory behavior in vivo using bioluminescent imaging. Cancer Res..

[26] Purvis J.E., Karhohs K.W., Mock C., Batchelor E., Loewer A., Lahav G. (2012). p53 dynamics control cell fate. Science.

[27] Batchelor E., Loewer A., Mock C., Lahav G. (2011). Stimulus-dependent dynamics of p53 in single cells. Mol. Syst. Biol..

[28] Sun T., Cui J. (2015). Dynamics of P53 in response to DNA damage: Mathematical modeling and perspective. Prog. Biophys. Mol. Biol..

[29] Batchelor E., Loewer A. (2017). Recent progress and open challenges in modeling p53 dynamics in single cells. Curr. Opin. Syst. Biol..

[30] Sun T., Yang W., Liu J., Shen P. (2011). Modeling the basal dynamics of p53 system. PLoS ONE.

[31] Zhang X.P., Liu F., Wang W. (2011). Two-phase dynamics of p53 in the DNA damage response. Proc. Natl. Acad. Sci. USA.

[32] Li Z., Ni M., Li J., Zhang Y., Ouyang Q., Tang C. (2011). Decision making of the p53 network: Death by integration. J. Theor. Biol..

[33] Tian X.J., Liu F., Zhang X.P., Li J., Wang W. (2012). A two-step mechanism for cell fate decision by coordination of nuclear and mitochondrial p53 activities. PLoS ONE.

[34] Chen X., Chen J., Gan S., Guan H., Zhou Y., Ouyang Q., Shi J. (2013). DNA damage strength modulates a bimodal switch of p53 dynamics for cell-fate control. BMC Biol..

[35] Zhang T., Brazhnik P., Tyson J.J. (2009). Computational analysis of dynamical responses to the intrinsic pathway of programmed cell death. Biophys. J..

[36] Zhuge C., Sun X., Chen Y., Lei J. (2016). PDCD5 functions as a regulator of p53 dynamics in the DNA damage response. J. Theor. Biol..

[37] Chong K.H., Samarasinghe S., Kulasiri D., Zheng J. (2019). Mathematical modelling of core regulatory mechanism in p53 protein that activates apoptotic switch. J. Theor. Biol..

[38] Toettcher J.E., Loewer A., Ostheimer G.J., Yaffe M.B., Tidor B., Lahav G. (2009). Distinct mechanisms act in concert to mediate cell cycle arrest. Proc. Natl. Acad. Sci. USA.

[39] Zhang X.P., Liu F., Cheng Z., Wang W. (2009). Cell fate decision mediated by p53 pulses. Proc. Natl. Acad. Sci. USA.

[40] Geva-Zatorsky N., Rosenfeld N., Itzkovitz S., Milo R., Sigal A., Dekel E., Yarnitzky T., Liron Y., Polak P., Lahav G. (2006). Oscillations and variability in the p53 system. Mol. Syst. Biol..

[41] Moore R., Ooi H.K., Kang T., Bleris L., Ma L. (2015). MiR-192-Mediated Positive Feedback Loop Controls the Robustness of Stress-Induced p53 Oscillations in Breast Cancer Cells. PLoS Comput. Biol..

[42] Elias J., Dimitrio L., Clairambault J., Natalini R. (2014). The dynamics of p53 in single cells: Physiologically based ODE and reaction-diffusion PDE models. Phys. Biol..

[43] Elias J., Dimitrio L., Clairambault J., Natalini R. (2014). The p53 protein and its molecular network: Modelling a missing link between DNA damage and cell fate. Biochim. Biophys. Acta.

[44] Wee K.B., Surana U., Aguda B.D. (2009). Oscillations of the p53-Akt network: Implications on cell survival and death. PLoS ONE.

[45] Tian X., Huang B., Zhang X.P., Lu M., Liu F., Onuchic J.N., Wang W. (2017). Modeling the response of a tumor-suppressive network to mitogenic and oncogenic signals. Proc. Natl. Acad. Sci. USA.

[46] Hat B., Kochanczyk M., Bogdal M.N., Lipniacki T. (2016). Feedbacks, Bifurcations, and Cell Fate Decision-Making in the p53 System. PLoS Comput. Biol..

[47] Mathupala S.P., Heese C., Pedersen P.L. (1997). Glucose catabolism in cancer cells. The type II hexokinase promoter contains functionally active response elements for the tumor suppressor p53. J. Biol. Chem..

[48] Schwartzenberg-Bar-Yoseph F., Armoni M., Karnieli E. (2004). The tumor suppressor p53 down-regulates glucose transporters *GLUT1* and *GLUT4* gene expression. Cancer Res..

[49] Su X., Gi Y.J., Chakravarti D., Chan I.L., Zhang A., Xia X., Tsai K.Y., Flores E.R. (2012). TAp63 is a master transcriptional regulator of lipid and glucose metabolism. Cell Metab..

[50] Liu B., Oltvai Z.N., Bayir H., Silverman G.A., Pak S.C., Perlmutter D.H., Bahar I. (2017). Quantitative assessment of cell fate decision between autophagy and apoptosis. Sci. Rep..

[51] Yu L., Lu M., Jia D., Ma J., Ben-Jacob E., Levine H., Kaipparettu B.A., Onuchic J.N. (2017). Modeling the Genetic Regulation of Cancer Metabolism: Interplay between Glycolysis and Oxidative Phosphorylation. Cancer Res..

[52] Markert E.K., Vazquez A. (2015). Mathematical models of cancer metabolism. Cancer Metab..

[53] Marin-Hernandez A., Gallardo-Perez J.C., Rodriguez-Enriquez S., Encalada R., Moreno-Sanchez R., Saavedra E. (2011). Modeling cancer glycolysis. Biochim. Biophys. Acta.

[54] Shestov A.A., Liu X., Ser Z., Cluntun A.A., Hung Y.P., Huang L., Kim D., Le A., Yellen G., Albeck J.G. (2014). Quantitative determinants of aerobic glycolysis identify flux through the enzyme GAPDH as a limiting step. Elife.

[55] Epstein T., Xu L., Gillies R.J., Gatenby R.A. (2014). Separation of metabolic supply and demand: Aerobic glycolysis as a normal physiological response to fluctuating energetic demands in the membrane. Cancer Metab..

[56] Gatenby R.A., Gawlinski E.T. (1996). A reaction-diffusion model of cancer invasion. Cancer Res..

[57] Gatenby R.A., Gawlinski E.T., Gmitro A.F., Kaylor B., Gillies R.J. (2006). Acid-mediated tumor invasion: A multidisciplinary study. Cancer Res..

[58] Robertson-Tessi M., Gillies R.J., Gatenby R.A., Anderson A.R. (2015). Impact of metabolic heterogeneity on tumor growth, invasion, and treatment outcomes. Cancer Res..

[59] Senft D., Ronai Z.A. (2016). Regulators of mitochondrial dynamics in cancer. Curr. Opin. Cell Biol..

[60] Achanta G., Sasaki R., Feng L., Carew J.S., Lu W., Pelicano H., Keating M.J., Huang P. (2005). Novel role of p53 in maintaining mitochondrial genetic stability through interaction with DNA Pol γ. EMBO J..

[61] Lebedeva M.A., Eaton J.S., Shadel G.S. (2009). Loss of p53 causes mitochondrial DNA depletion and altered mitochondrial reactive oxygen species homeostasis. Biochim. Biophys. Acta.

[62] Bakhanashvili M., Grinberg S., Bonda E., Simon A.J., Moshitch-Moshkovitz S., Rahav G. (2008). p53 in mitochondria enhances the accuracy of DNA synthesis. Cell Death Differ..

[63] Park J.H., Zhuang J., Li J., Hwang P.M. (2016). p53 as guardian of the mitochondrial genome. FEBS Lett..

[64] Patel P.K., Shirihai O., Huang K.C. (2013). Optimal dynamics for quality control in spatially distributed mitochondrial networks. PLoS Comput. Biol..

[65] Zamponi N., Zamponi E., Cannas S.A., Billoni O.V., Helguera P.R., Chialvo D.R. (2018). Mitochondrial network complexity emerges from fission/fusion dynamics. Sci. Rep..

[66] Capps G.J., Samuels D.C., Chinnery P.F. (2003). A model of the nuclear control of mitochondrial DNA replication. J. Theor. Biol..

[67] Kowald A., Dawson M., Kirkwood T.B. (2014). Mitochondrial mutations and ageing: Can mitochondrial deletion mutants accumulate via a size based replication advantage?. J. Theor. Biol..

[68] Sukhorukov V.M., Dikov D., Reichert A.S., Meyer-Hermann M. (2012). Emergence of the mitochondrial reticulum from fission and fusion dynamics. PLoS Comput. Biol..

[69] Mouli P.K., Twig G., Shirihai O.S. (2009). Frequency and selectivity of mitochondrial fusion are key to its quality maintenance function. Biophys. J..

[70] Tam Z.Y., Gruber J., Halliwell B., Gunawan R. (2013). Mathematical modeling of the role of mitochondrial fusion and fission in mitochondrial DNA maintenance. PLoS ONE.

[71] Gillespie D.T. (2007). Stochastic simulation of chemical kinetics. Annu. Rev. Phys. Chem..

[72] Kim M.P., Lozano G. (2018). Mutant p53 partners in crime. Cell Death Differ..

